# A Machine Learning Method for Identifying Lung Cancer Based on Routine Blood Indices: Qualitative Feasibility Study

**DOI:** 10.2196/13476

**Published:** 2019-08-15

**Authors:** Jiangpeng Wu, Xiangyi Zan, Liping Gao, Jianhong Zhao, Jing Fan, Hengxue Shi, Yixin Wan, E Yu, Shuyan Li, Xiaodong Xie

**Affiliations:** 1 State Key Laboratory of Applied Organic Chemistry Lanzhou University Lanzhou China; 2 College of Chemistry and Chemical Engineering Lanzhou University Lanzhou China; 3 Department of Pneumology Lanzhou University Second Hospital Lanzhou China; 4 Department of Radiology Lanzhou University Second Hospital Lanzhou China; 5 National Demonstration Centre for Experimental Chemistry Education Lanzhou University Lanzhou China; 6 School of Basic Medical Science Lanzhou University Lanzhou China

**Keywords:** lung cancer identification, routine blood indices, Random Forest

## Abstract

**Background:**

Liquid biopsies based on blood samples have been widely accepted as a diagnostic and monitoring tool for cancers, but extremely high sensitivity is frequently needed due to the very low levels of the specially selected DNA, RNA, or protein biomarkers that are released into blood. However, routine blood indices tests are frequently ordered by physicians, as they are easy to perform and are cost effective. In addition, machine learning is broadly accepted for its ability to decipher complicated connections between multiple sets of test data and diseases.

**Objective:**

The aim of this study is to discover the potential association between lung cancer and routine blood indices and thereby help clinicians and patients to identify lung cancer based on these routine tests.

**Methods:**

The machine learning method known as Random Forest was adopted to build an identification model between routine blood indices and lung cancer that would determine if they were potentially linked. Ten-fold cross-validation and further tests were utilized to evaluate the reliability of the identification model.

**Results:**

In total, 277 patients with 49 types of routine blood indices were included in this study, including 183 patients with lung cancer and 94 patients without lung cancer. Throughout the course of the study, there was correlation found between the combination of 19 types of routine blood indices and lung cancer. Lung cancer patients could be identified from other patients, especially those with tuberculosis (which usually has similar clinical symptoms to lung cancer), with a sensitivity, specificity and total accuracy of 96.3%, 94.97% and 95.7% for the cross-validation results, respectively. This identification method is called the routine blood indices model for lung cancer, and it promises to be of help as a tool for both clinicians and patients for the identification of lung cancer based on routine blood indices.

**Conclusions:**

Lung cancer can be identified based on the combination of 19 types of routine blood indices, which implies that artificial intelligence can find the connections between a disease and the fundamental indices of blood, which could reduce the necessity of costly, elaborate blood test techniques for this purpose. It may also be possible that the combination of multiple indices obtained from routine blood tests may be connected to other diseases as well.

## Introduction

Using liquid biopsies based on blood tests is a promising method to achieve noninvasive diagnosis of cancers, but it is also currently a challenge in oncology [1-3]. The main approach for this technique involves the detection of circulating tumor DNAs (ctDNA) [4-6] or specific protein biomarkers [7,8] in plasma. Other cancer biomarkers, such as metabolites [9,10], autoantibodies [11,12], antigens [13,14], microRNAs [15-17], long noncoding RNAs [18,19], and methylated DNAs [3,20,21] were also used. The advantages of this approach include its convenience, and that it is both noninvasive and effective for helping physicians to decide or adjust the treatment schedule for a patient [5,22]. However, its proper usage is still being debated, in part because of its varied results among different patients but also due to its relatively low sensitivity and specificity [7,17,22,23].

Cancers that can be detected with liquid biopsy methods include breast [10], stomach [24], liver [18], pancreas [19], esophagus [14], prostate [17], colorectum [25], laryngeal [9], ovary [26] and lung [27] cancers. Cohen et al even demonstrated the possibility of identifying eight common cancer types simultaneously using blood biopsy, including lung, ovary, liver, stomach, pancreas, esophagus, colorectum and breast cancer, based on a multi-analyte blood test [1]. Among these cancers, lung cancer has a consistently high morbidity and mortality rate compared to all other types of cancers [28], and it has become the leading cause of cancer death worldwide [29]. Therefore, liquid biopsy studies on lung cancer, especially using multiple biomarkers, have attracted a lot of attention [16]. For instance, Leng et al used the integrity of cell-free DNAs to distinguish lung cancer patients from healthy ones with a sensitivity of 79.2% and a specificity of 67.3% [30]. Li et al used a combination of 13 protein biomarkers as a classifier to distinguish lung cancer and reached a sensitivity of 93% [31]. Chen et al utilized 10 serum microRNAs as biomarkers to identify lung cancer and achieved a sensitivity of 93% as well as a specificity of 90% [32]. These results suggest that a combination of multiple biomarkers performs better than testing for a single marker.

Meanwhile, misdiagnosis of lung cancer and tuberculosis occurs frequently in clinical situations [33] due to some misleading images obtained by computed tomography (CT) scans. This is one of the most common detection approaches for lung cancer in the clinic, along with tissue biopsies, as CT scans can detect a smaller nodule and find hidden areas when detecting lung cancer. However, they aren’t specific enough to identify lung cancer from benign nodules and tuberculosis [34]. Therefore, patients who are not immediately found to have lung cancer usually undergo unnecessary tissue biopsies, such as needle biopsy, bronchoscopy, thoracoscopy, mediastinoscopy or thoracotomy [35]. Aiming at this problem, Leng et al tried to use DNA biomarkers to distinguish lung cancer from tuberculosis and got an 82.9% specificity and a barely satisfactory 55.7% sensitivity [30].

In this work, inspired both by the fact that multi-analyte blood tests can reveal greater correlation between complicated connections, and that comprehensive consideration of multiple factors may also mitigate the effects of variation between individual patients, we tried to find the connection between the results of routine blood examinations and serious diseases. Although none of the blood test data for a single factor was proven to be the sole indicator of lung cancer, it was found that a combination of 19 routine blood biochemical indices were highly related as indicators of lung cancer, based on the Random Forest method [36]. This approach presented a chance to classify lung cancer through the use of a cross-validation set and a test set, with tuberculosis samples included. To the best of our knowledge, this is the first time that a combination of routine blood biochemical indices is presented for its capability to well distinguish lung cancer, especially from tuberculosis.

## Methods

### Source of Materials

Data from routine blood tests were collected from the Second Hospital of Lanzhou University. A total of 277 patients with 49 types of routine blood indices were included in this study, including 183 patients whose lung cancer was diagnosed by tissue biopsies as positive samples and another 94 patients, without lung cancer, as negative samples. These patients ranged from 20 to 81 years of age, and general information about their data sets can be accessed in Table 1 (for detailed information about these patients, including sex, age, smoking status, cancer stage and blood indices, see Multimedia Appendix 1). It should be noted that among the 94 negative patients, 51 with tuberculosis were specifically included since there is a high false positive rate in using CT scans to distinguish lung cancer from tuberculosis. Tuberculosis patients were carefully diagnosed with a combination of CT images and clinical symptoms by an experienced clinician. The other patients in the negative group just went to the hospital for routine physical examinations and were not diagnosed with any lung tissue–related diseases. All of the samples were collected from unrelated patients. The Lanzhou University Ethics Committee granted approval of this study and each participant signed an informed consent form after receiving a verbal explanation of the study.

After collection, the data were randomly split into a training set and a test set with a ratio of about 4 to 1. The training set included 222 patients and was constructed with 149 lung cancer samples, 37 tuberculosis samples, and 36 other samples, and then the remaining 55 samples were assigned to the test set.

**Table 1:** General demographic information on the test set and the training set (N=277).

Characteristic		Training set			Test set		
		Lung cancer (n=149)	Tuberculosis (n=37)	Other (n=36)	Lung cancer (n=34)	Tuberculosis (n=14)	Other (n=7)
**Gender, n**							
	Male	110	37	12	22	5	5
	Female	39	20	24	12	9	2
Median age (range)		60 (27-81)	46 (20-79)	55 (30-78)	58 (38-79)	52 (20-78)	62 (49-68)
Smokers, n		44	2	2	5	0	1

### Machine Learning Method

The Random Forest method (RF) [36] was adopted here to build the final classification model. RF is a very powerful and practical classifier that can use multiple trees to train an AI to predict samples, and it has been extensively employed in the fields of chemometrics and bioinformatics [37]. There are two main advantages to the RF method which are that, first, it can use an out of the bag set to monitor errors, strengths, and correlation [38], and second, it can measure variable importance through permutation. The RF method can handle high-dimensional data and approach the best predictor for them by further decreasing the dimensions of feature space and discovering rigorous feature numbers. For this algorithm, the two most important parameters were the tree number (ntree) and the number of randomly selected features to split at each node (mtry), which needed to be adjusted to get the best classification model. In this work, we at first made use of the entire set of indices to establish an RF classification prediction model on the basis of the 10-fold cross-validation. For each index, the importance of its association with the prediction target was demonstrated in this procedure. Then, based on increasing the number of top-ranking indices, the RF model was built with adjusted parameters. The initial value of ntree was 100, which increased by 100 until it reached 1500. The value of mtry was set to 2-10 with a step of 1. Finally, we chose the most suitable model with the fewest number of top-ranking indices but with a similar prediction performance compared to the entire index space. Then, the 19 top-ranking indices with ntree and mtry values of 1300 and 9, respectively, were selected for the final model. This selection process also helped us to locate the key indices for predicting lung cancer. RF was executed by applying the Random Forest package of R.

### Validation Method

Both internal cross-validation and further tests were adopted to obtain a reliable classifier for lung cancer. The entire modelling process, including feature ranking, RF parameter adjusting, and final model selection, was performed based only on the training set using 10-fold cross-validation. The presplitting test set for further testing of the built model was not involved in any of these model-building processes, as emphasized by Smialowski et al [39]. Ten-fold cross-validation is employed to randomly divide the training set into 10 nonoverlapping parts, one of which is used as an internal test set while the rest are used as the training set. This process is repeated 10 times so that all samples can be used as an internal test set once. The circular work thus facilitates the potential establishment of a stable classification model for predicting lung cancer. The average results were obtained after 10 runs of the circular process as the final 10-fold cross-validation result.

Five frequently used indicators were adopted here to evaluate the final performance of the routine blood indices model for lung cancer (RBLC) method, including sensitivity (Sens), specificity (Spec), accuracy (ACC), Matthews correlation coefficient (MCC), and the area under the curve (AUC), where TP, TN, FP, and FN stand for true positive, true negative, false positive, and false negative, respectively.

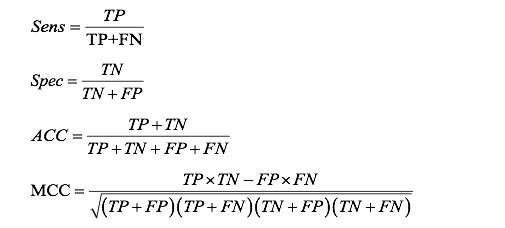



The receiver operating characteristic (ROC) curve is a composite indicator and a graphical plot for the continuous variables of Sens and Spec, with Sens as the y-axis and 1–Spec as the x-axis. One characteristic of the ROC curve is that it could remain unchanged if the positive and negative samples are out of balance in the test set.

AUC is the area under the ROC curve, and it can range from a value of 0 to a value of 1. The closer the AUC is to 1, the better the prediction performance of lung cancer. It is one of the main evaluation indices for a binary classifier system.

## Results

### Model Selection

Routine blood tests listed in Multimedia Appendix 1 are easy to perform and low cost, but no direct connection between these routine blood tests and the diagnosis of cancers has been found and used in clinical trials yet. This is one of the most important reasons for a surge in interest in finding new biomarkers for cancers. Recent research has indicated some comprehensive connections between certain symptoms and some disorders, such as Axelsson et al demonstrating the facial cues of sick people [40]. However, these studies left unanswered the question of if it was possible to use machine learning methods to find any connection between cancer and these routine blood indices.

**Figure 1:**
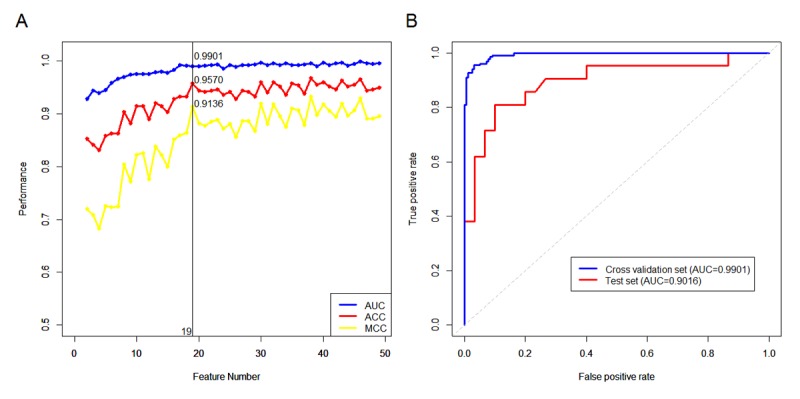
Classification performance of the RBLC model. (A) Cross-validation results of models which were built on top ranking features. (B) ROC curves and the corresponding AUCs for the cross-validation on the training set and for the test set. RBLC: routine blood indices model for lung cancer; ROC: receiver operating characteristic; AUC: area under the curve; ACC: accuracy; MCC: Matthews correlation coefficient.

To answer that question in this study, we used routine blood and biochemical test data that can be measured by common chemistry analyzers, with a cost of approximately $10-20 for each sample, to determine their correlation with lung cancer. Surprisingly, positive correlation was found with a simple Random Forest (RF) test method, with 19 blood indices enough to prove correlation. With the data set we used, an MCC of 91.36%, ACC of 95.7% (Figure 1A) and AUC of 99.01% (Figure 1B) were attained. The detailed information about these 19 indices, such as their typical values, units and biological meanings, can be found in in Table 2. The model that was constructed is referred to as RBLC.

In fact, 19 indices are equivalent to a critical point (Figure 1A). The principle of selecting the number of features was to use the minimum features possible to achieve a comparable prediction performance as the entire feature space. The fewer features that a model consists of, the less probability it gets an overfitting problem. If the number of features was increased from 19 to 38, many features would be unnecessary because its results would be comparable to the previous predictive performance. Therefore, in our opinion it is a better choice that the final model has only 19 features, to not only establish a simple, efficient and robust classification model, but also to avoid excessive waste of blood test procedures and save diagnosis time.

**Figure 2:**
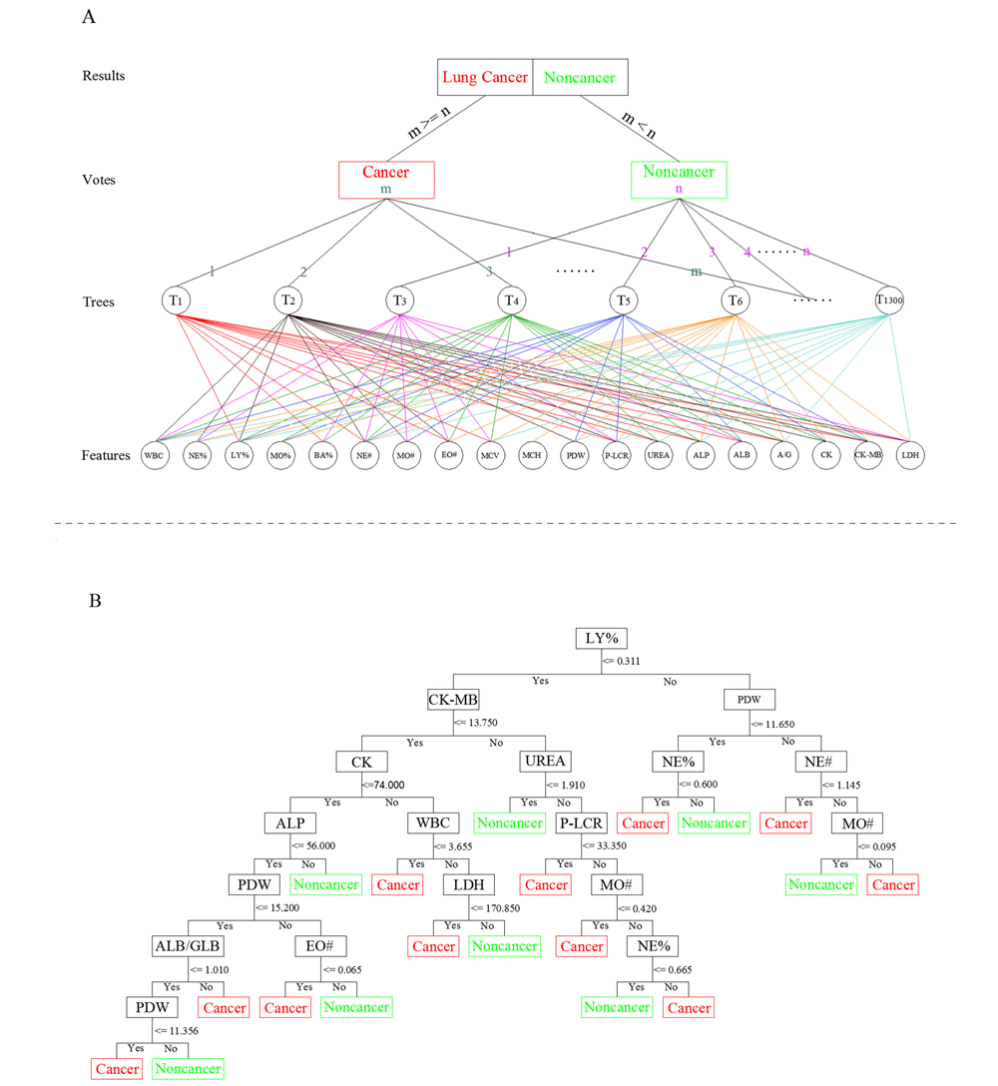
The detailed forest structure for the RBLC model. (A) The general structure of the voting strategy of the RBLC model. (B) The independent decision rulings for different blood indices for the first tree (T1) in (A). T: tree; WBC: white blood cell count; NE%: neutrophil granulocyte ratio; LY%: lymphocyte ratio; MO%: monocyte ratio; BA%: basophil ratio; NE#: neutrophilic granulocytes; MO#: monocytes; EO#: eosinophil cells; MCV: mean corpuscular volume; MCH: mean corpuscular hemoglobin; PDW: platelet distribution width; P-LCR: platelet large cell ratio; UREA: carbamide; ALP: alkaline phosphatase; ALB: albumin; A/G: albumin/globulin; CK: creatine kinase; CK-MB: creatine kinase isoenzymes; LDH: lactate dehydrogenase.

The detailed forest structure for the RBLC model is illustrated in Figure 2. Each tree in the forest votes for the major classification based on different combinations of blood indices, and the majority of votes results in the final classification of the RBLC model (Figure 2A). In addition, each node in each tree votes for the classification, upon independent decision rule, for each different blood index, and hence deduces a final vote for a single tree (Figure 2B). This model achieved not only a great improvement in sensitivity and specificity but also high precision prediction performance, such that the sensitivity, specificity, and accuracy scores were all greater than 85% in the test set, with values of 85.71%, 90%, 88.24%, respectively. The MCC value and AUC for the test set also got 75.71% and 90.16%, respectively. These results indicate that this RBLC method has the optimum and stable prediction performance needed to distinguish lung cancer from tuberculosis and other samples.

**Table 2:** Top-ranking blood indices for the identification of lung cancer.

Rank	Index	Reference range
1	Basophil ratio	0.00-0.01
2	Creatine kinase isoenzymes (U/L)	0.0-25.0
3	Platelet large cell ratio (%)	17.0-45.0
4	Albumin (g/L)	30.0-55.0
5	Platelet distribution width (fl)	9.0-17.0
6	Neutrophilic granulocytes (10^9^/L)	2.00-7.00
7	White blood cell count (10^9^/L)	4.00-10.00
8	Albumin/Globulin ratio	1.10-2.50
9	Monocytes (10^9^/L)	0.12-1.20
10	Monocyte ratio	0.03-0.08
11	Lymphocyte ratio	0.20-0.40
12	Neutrophil granulocyte ratio	0.50-0.70
13	Lactate dehydrogenase (U/L)	0.0-240.0
14	Carbamide (mmol/L)	1.80-8.00
15	Eosinophil cells (10^9^/L)	0.02-0.50
16	Mean corpuscular volume (fl)	80.0-100.0
17	Alkaline phosphatase (U/L)	0.0-120.0
18	Mean corpuscular hemoglobin (pg)	27.0-34.0
19	Creatine kinase (U/L)	0-195

### Clinical Relevance

To confirm the efficiency, reliability, and repeatability of the RBLC model, 34 serial blood samples from 15 additional patients were also included in the study (detailed information, including the patients’ sex, age, smoking status, cancer stage and blood data, is listed in Multimedia Appendix 2). Five of these patients were diagnosed with lung cancer by lung tissue biopsy when they got their first blood examination, and then serial blood tests were performed afterward either weekly or monthly (for 13 samples in all). Of the blood samples collected, 11 were from 5 patients who were diagnosed with tuberculosis (without lung cancer) and 10 were from 5 patients who were diagnosed with neither lung cancer nor tuberculosis. These samples were used as the negative controls. Among these samples, 12/13 with lung cancer, 8/11 with tuberculosis and 9/10 healthy samples were accurately identified. Overall, the sensitivity reached 92.31%, the specificity reached 80.95%, and the total accuracy reached 85.29%. This result for the additional serial data is fairly consistent with the results of the single-sample test in the test set, which further proves the reliability and stability of the RBLC model. More importantly, it appears to be able to distinguish tuberculosis and lung cancer.

### Web Server of Routine Blood Indices Model for Lung Cancer Method

**Figure 3:**
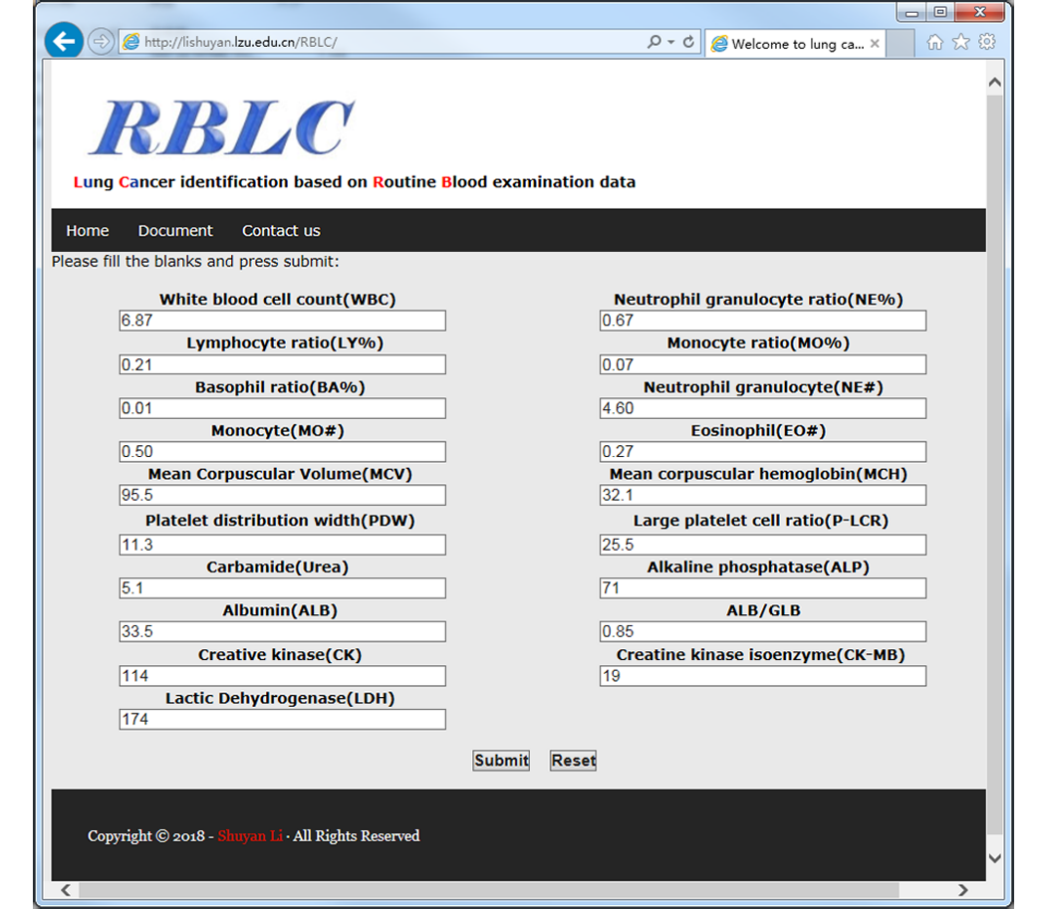
Web page of the RBLC tool for convenient usage online. RBLC: routine blood indices model for lung cancer; ALB/GLB: albumin/globulin.

A user-friendly web server is available online to use the RBLC method [41]. Users can input the 19 key features from a routine blood examination and blood biochemical examination into the corresponding text boxes on the web page (Figure 3) and then press the Submit button. After calculation and analysis of the outputs of the sample, the results page will display whether the input is considered a sample with lung cancer or not.

## Discussion

### Overview

The performance of the RBLC method was compared to other commonly used identification methods of lung cancer and ended up showing a favorable result, and then, the association of these selected key routine blood indices with lung cancer was analyzed and further confirmed.

### Performance Comparison

With regard to other identification methods, CT scans are a common tool for the detection of lung cancer. For instance, the National Lung Screening Trial (NLST) recommends the use of CT scans to help diagnose patients at high risk for lung cancer. The NLST also demonstrated that mortality could be reduced by 20% using CT screening, with a specificity of 72.6% [42]. However, the low specificity of CT may expose patients to anxiety and unnecessary further examinations.

**Table 3:** Comparison of the performance of different methods for predicting lung cancer on cross-validation.

Prediction method	Sample size	Sensitivity, %	Specificity, %	Area under the curve
RBLC^a^	226	96.30	94.97	0.99
Protein biomarker [31]	143	93.00	45.00	N/A^b^
RNA biomarker [32]	310	93.00	90.00	0.97
DNA biomarker [30]	318	79.20	67.30	0.75
Computed tomography scans [43]	N/A	94.40	72.60	N/A

^a^RBLC: routine blood indices model for lung cancer.

^b^N/A: not applicable.

Currently, biomarker analysis is another prevalent technique for detecting lung cancer in high-risk populations. Different lung cancer–related components are ideal biomarkers for the detection of lung cancer. The protein, DNA, and RNA referenced in Table 3 are the latest biomarkers to be developed. Compared to these other methods, the RBLC model demonstrates satisfactory performance in terms of sensitivity, specificity, and AUC, and it is much easier to perform. It is noteworthy that 94.74% of early stage (stage I/II) patients were distinguished by RBLC (see Multimedia Appendix 1), which implies it has further potential for application for identification of early-stage lung cancer.

### Key Blood Indices Analysis

Detailed information for the selected key indices for the RBLC model was shown in Table 2, and these indices were listed in decreasing order of importance. Afterward, all the values of these indices were normalized on a scale going from 0 to 1, and then the average values for both positive and negative samples were shown in Table 4. The *P* values within the table were determined using two-tailed *t* tests.

Among these key indices, the relationship between lactate dehydrogenase (LDH) and lung cancer has been discussed extensively [44]. The expression of LDH not only increases points in glucose metabolism progression, but research has also shown it has a strong association with lung cancer [45]. In this work, the LDH levels of blood samples from lung cancer patients was significantly different from that of negative samples (*P*<.001), which is consistent with previous studies as well.

**Table 4:** Feature comparison of lung cancer and other samples.

Feature	Negative sample	Positive sample (lung cancer)	*P* value
White blood cell count	0.1986	0.3088	<.001
Neutrophil-granulocyte ratio	0.4257	0.6502	<.001
Lymphocyte ratio	0.5298	0.3232	<.001
Monocyte ratio	0.4319	0.3970	.20
Basophil ratio	0.2555	0.1242	<.001
Neutrophilic granulocytes	0.1839	0.2808	<.001
Monocytes	0.2795	0.384	<.001
Eosinophil cells	0.3236	0.0833	<.001
Mean corpuscular volume	0.6808	0.5453	<.001
Mean corpuscular hemoglobin	0.6545	0.5983	.008
Platelet distribution width	0.5765	0.6337	.03
Platelet large cell ratio	0.5081	0.4010	<.001
Carbamide	0.4181	0.3197	<.001
Alkaline phosphatase	0.4138	0.1366	<.001
Albumin	0.5757	0.5574	.52
Albumin/globulin	0.3917	0.4155	.46
Creatine kinase	0.1103	0.0867	.19
Creatine kinase Isoenzymes	0.3557	0.2014	<.001
Lactate dehydrogenase	0.5441	0.1462	<.001

In addition, white blood cell count (WBC) is one of the most commonly used, nonspecific markers of inflammation [46]. Chronic bronchitis in a patient would be accompanied by an increase in their WBC, but the association between lung cancer risk and elevated WBC goes beyond preexisting, increased levels [47]. In addition, most tumors are surrounded by inflammatory cells which play an important role in the pathogenesis of cancer by recruiting immune cells that promote survival of the tumor [48]. Our results, like other studies, show a positive association between WBC and lung cancer, in which lung cancer patients have a relatively higher average WBC than negative samples (*P*<.001), although most of the indicators are in the normal clinical range. In previous studies, researchers mainly focused on the value of the neutrophil to lymphocyte ratio as a predictor of lung cancer [49], while neutrophil-granulocyte ratio (NE%) wasn’t really considered to be an independent index. The NE% of lung cancer has an obvious difference compared with negative samples (*P*<.001) in our work, which may be of practical importance.

Research on eosinophil cells (EO#) associated with lung cancer is rarely reported. The significant difference in the EO# between lung cancer samples (*P*<.001) and negative samples is indicated in this study as well. There is a common view that paraneoplastic processes and distant metastases (to the bone marrow) will increase EO# to some extent [50]. Alkaline phosphatase (ALP) is reported to be associated with cancer metastasis in the literature [51], and it was also a critical index for identifying lung cancer and negative samples in our analysis.

Although creatine kinase isoenzymes (CK-MB) have a good specificity for diagnosis of myocardial infarction, related reports have indicated that the presence of malignant tumors can cause a significant distinction in CK-MB levels [52]. Our study also suggested that CK-MB (*P*<.001) has a significantly different average value in lung cancer compared to negative samples.

### Conclusion

All of above the results demonstrate that the blood indices we selected were related to lung cancer to some extent, but none of them solely exhibits a clear connection and can be used for diagnostic purposes. With the aid of machine learning, through a combination of multiple test items and connections between the complicated patterns of these blood indices, specific diseases may be distinguished. The identification performance of the RBLC model for lung cancer is rather encouraging, as shown in Table 3. We thus believe that machine learning can reveal the complicated correlation between routine blood test data and other serious diseases, which is currently a case of ongoing research in our group.

## Appendix

Multimedia Appendix 1 Detailed information of the samples for RBLC modeling and validation.

Multimedia Appendix 2 Detailed information of the samples for further clinical relevance evaluation.

## Abbreviations

ACC: accuracy
ALP: alkaline phosphatase
AUC: area under the curve
CK-MB: creatine kinase isoenzymes
ctDNA: circulating tumor DNA
CT: computed tomography
EO#: eosinophil cells
FN: false negative
FP: false positive
LDH: lactate dehydrogenase
MCC: Matthews correlation coefficient
mtry: number of randomly selected features to split at each node
NE%: neutrophil granulocyte ratio
NLST: National Lung Screening Trial
ntree: tree number
RBLC: routine blood indices model for lung cancer
RF: Random Forest method
ROC: receiver operating characteristic
Sens: sensitivity
Spec: specificity
TN: true negative
TP: true positive
WBC: white blood cell count
